# Advances in Skin Regeneration Using Tissue Engineering

**DOI:** 10.3390/ijms18040789

**Published:** 2017-04-07

**Authors:** Komal Vig, Atul Chaudhari, Shweta Tripathi, Saurabh Dixit, Rajnish Sahu, Shreekumar Pillai, Vida A. Dennis, Shree R. Singh

**Affiliations:** Center for Nanobiotechnology Research, Alabama State University, 1627 Harris Way, Montgomery, AL 36104, USA; achaudhari@alasu.edu (A.C.); tripshweta@gmail.com (S.T.); sdixit@alasu.edu (S.D.); sahu.rajnish@gmail.com (R.S.); spillai@alasu.edu (S.P.); vdennis@alasu.edu (V.A.D.); ssingh@alasu.edu (S.R.S.)

**Keywords:** tissue engineering, skin regeneration, skin substitutes

## Abstract

Tissue engineered skin substitutes for wound healing have evolved tremendously over the last couple of years. New advances have been made toward developing skin substitutes made up of artificial and natural materials. Engineered skin substitutes are developed from acellular materials or can be synthesized from autologous, allograft, xenogenic, or synthetic sources. Each of these engineered skin substitutes has their advantages and disadvantages. However, to this date, a complete functional skin substitute is not available, and research is continuing to develop a competent full thickness skin substitute product that can vascularize rapidly. There is also a need to redesign the currently available substitutes to make them user friendly, commercially affordable, and viable with longer shelf life. The present review focuses on providing an overview of advances in the field of tissue engineered skin substitute development, the availability of various types, and their application.

## 1. Introduction

Skin is the barrier between the internal and external environment and is the largest organ of the human body. Due to the presence of stem cells, the wounded epidermis is able to stimulate self-regeneration. However, in case of deep injuries and burns, the process of healing is not adequate, thus leading to a chronic wound. Any loss of full-thickness skin more than 4 cm diameter needs grafting for its treatment [1]. Additionally, many chronic wounds fail to heal, which can lead to amputations and mortality. These can also escalate health care costs. Surgical procedures available for skin healing often have limited availability of healthy donor tissue. The use of foreign tissue does provide a substitute; however, it also poses a risk of infection and immune rejection.

Tissue engineered skin substitutes are upcoming alternatives to traditional wound healing strategies and tissue regeneration. Among the tissue engineered organs, skin was the first engineered organ that went from laboratory research to patient care [2]. Over recent decades, various bioengineered and synthetic substitutes have been developed, which are generally positioned within the injury and provide the barrier function along with protection against microorganisms, reduction of pain in wounds, and promotion of wound healing by tissue regeneration [3,4,5].

The field of tissue engineering is an evolving field, and newer procedures are being developed and adopted to generate skin substitutes for clinical applications, even though the high cost for developing these substitutes is a major concern. The current review highlights the progress in the field of tissue-engineering and investigates various commercially available skin substitutes along with their advantages and shortcomings.

## 2. Anatomy of Skin

**Figure 1:**
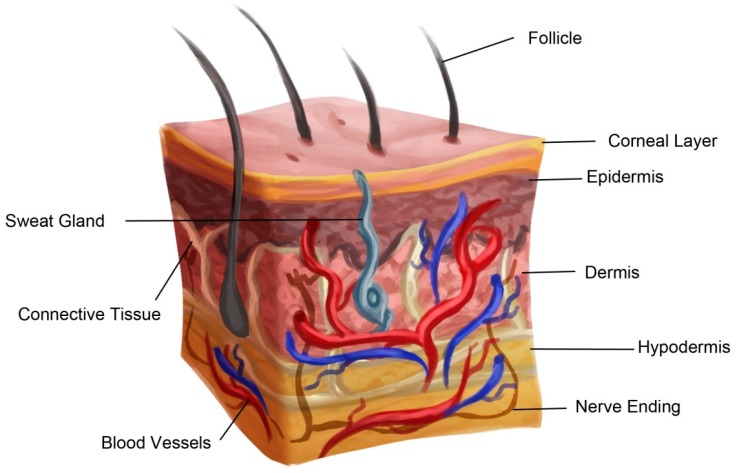
Normal skin structure.

The skin is the largest organ of the body. It compromises 15% of the total adult body weight and provides protection against external physical, chemical, and biologic agents. It also plays role in thermoregulation. Skin consists of three layers: epidermis, dermis, and hypodermis (subcutaneous layer) (Figure 1). The outermost portion of the epidermis, the Stratum corneum, is comparatively more waterproof than other layers and thus inhibits entry of pathogens and other foreign substances into the body. It is multi-layered, with cells extending from the basement membrane to the dermis. The basement membrane contains progenitor cells, which differentiate into keratinocytes. Keratinocytes differentiate and mature as they move towards the surface of skin. The keratinized layer of dead cells at the skin provides barrier properties to skin [6]. Skin color pigment, melanin produced by melanocytes, are present in basal layer of the epidermis. Melanin also helps to filter out ultraviolet radiation from sunlight. The epidermis also has Langerhans cells, which are part of the skin’s immune system.

The dermis is the thickest of the three layers of skin and is present just below the epidermis. It is a connective tissue made of extra cellular matrix (ECM), fibroblasts, vascular endothelial cells, along with hair follicles, sweat glands, sebaceous glands, blood vessels and nerve endings [6]. Fibroblasts are the main population of the dermis, which secretes collagen and elastin and thus provides mechanical strength and elasticity to the skin.

Below the dermis is the adipose tissue hypodermis layer. It provides insulation and cushioning between the skin and skeletal structures, such as bone and muscle. It also serves as an energy storage area [6].

## 3. Wounds

Each year in the United State alone, there are 70,000 burn injuries [7] and 600,000–1,500,000 venous leg ulcers injuries [8]. The treatment of wounds costs nearly $20 billion annually in the US [9].

Wound treatment mainly includes quick closure of wound to reinstate the barrier function of skin and prevent infection, along with pain suppression and functional recovery. Wound healing in skin is initiated by fibroblasts, which deposit a temporary tissue matrix. This is followed by inflammation and re-epithelization by keratinocytes. Further wound revascularization along with ECM deposition, angiogenesis, and remodeling takes place for wound healing and restoration [6,10].

Every trauma often results in acute skin wounds. After the repair process, these wounds often lead to a benign scar if not treated in time [11]. However, depending upon the extent of the wound area and/or depth, sometime, a chronic or non-healing wound can also result. Wounds that do not proceed through orderly and timely restitution of structural and functional integrity often results in chronic wounds. Generally, vascular insufficiency, local-pressure effects, and conditions like diabetes mellitus, along with compromised nutritional or immunological status, are the major causes of non-healing skin wounds. Healing capability of a wound is affected by aging, which leads to decreased strength and elasticity of skin, decreased blood flow to the extremities, and psychological stress [12,13].

## 4. Process of Skin Wound Healing

After a skin injury, the damaged tissue is restored through coordinated signaling that constitutes the cutaneous healing response. This cutaneous response proceeds in three phases: the inflammatory phase, proliferation phase, and maturation phase. The inflammatory phase of wound healing typically lasts for the initial four days [14] and begins with coagulation of blood resulting in a blood clot that provides a temporary shield against pathogens as well as fluid loss. It is followed by increased blood flow in the areas adjacent to the wound trailed by swelling and redness due to increased vascular permeability by local inflammatory agents (activated complement, histamine, etc.) leading to plasma extravasation and generation of a fibrin matrix [6]. Neutrophils, monocytes, and other immunocompetent cells invade this matrix to remove dead tissue and control infection.

After the inflammatory phase, between 5–20 days, proliferation of vascular endothelial cells and fibroblasts is promoted due to secretion of growth factors by inflammatory cells. Fibrin matrix is gradually replaced by collagen secreted by fibroblasts. Fibroblasts can differentiate into myofibroblasts expressing actin resulting in contraction and reduction of the wound area. Adjacent healthy tissue, as well as endothelial progenitors, initiate angiogenesis, which leads to invasion of vascular endothelial cells and capillaries, resulting in the formation of “granulation tissue” [6]. It is followed by migration of keratinocytes from the edges of the wound to the surface of the granulation tissue, below the blood clot [14].

During the last phase of maturation, re-epithelialization of the wound takes place along with dermis regaining its tensile strength. However, the scar continues to remodel over several months to years [6].

## 5. Conventional Treatments for Wound Healing

Different strategies have been employed for wound treatments, which are discussed below:

### 5.1. Skin Grafting with Autograft

Due to lack of keratinocytes to reform the epithelium, deeper dermal wounds heal slowly and inadequately. Skin grafting with an “autograft” is recommended for such wounds [15]. Skin autograft technique was used in India in premodern times [16]. A thin layer of skin that includes the full epidermis and a portion of the dermis, known as split-thickness graft, is shaved from the donor site, such as inner thighs and buttocks, using a dermatome, and is then placed on the wound site [17]. The healing of wound depends on thickness of the underlying dermis in the graft. Thick dermis leads to faster healing and better cosmetic and aesthetic aspect of the healed wound. Donor sites can be reharvested a few times after healing, as the sites thin down due to lack of regeneration of the dermis after each harvest. Since an autograft is derived from the patient’s own tissue, there is no risk of rejection [15].

### 5.2. Skin Allografts

A method to overcome the limitation of donor tissue for skin grafting with an autograft is to use allografts. Human skin allograft clinical use was first described in the manuscript of Branca of Sicily in 1503. Skin allografts have been used since World War II.

Cadavers are a good source of allografts. As cadavers are stored frozen in skin banks, they can be used as needed [18]. Allografts from cadavers are used extensively in burn wound management in many burn centers all over the world. Skin allografts can also be obtained from living donors.

A viable skin allograft can revascularize like autologous split skin grafts. Though allografts not only provide a barrier along with promoting angiogenesis, providing growth factors, and essential cytokines for wound healing [19], they also serve as a temporary cover due to immunogenic rejection by the host’s immune system [15,20] and viral transmission (e.g., hepatitis B and C or HIV). Application of human skin allograft guards underlying tissue while allowing granulation tissue formation and wound contraction. Subsequent skin autograft and secondary skin contraction and epithelialization provide satisfactory wound closure [21].

### 5.3. Xenografts

A surgical graft from one species to another dissimilar species is known as xenograft. Skin substitutes harvested from the animals that are used on human wounds constitute xenograft and can be used as temporary grafts for human wounds. Xenograft fuses exogenous collagen into the wound, thus assisting dermal regeneration. These xenografts get absorbed as the wound heals, thus making them perfect for surgical wounds [22]. Skin xenograft was reported in the Papyrus of Ebers in the 15th century BC [19]. The earliest reported xenograft was with frog skin in 1500 BC. These are usually employed as temporary coverage [23]. Most commonly used xenografts are from porcine skin, which is often used in burn care [19].

### 5.4. Amnion

Since 1910, amnion has been used as a dressing for burns [19]. Amnion is primarily used for partial thickness burns, such as facial burns, and is one of the most effective biological skin substitutes [24]. Amnion is also used in sandwich grafting technique. Amnion is usually collected from the placentae of selected and screened donors and preserved for further use. It reduces loss of protein, electrolytes, and fluids, minimizes pain, decreases the risk infection, and accelerates wound healing. In a recent clinical trial, amnion dressing was used for the donor site on 32 patients, which resulted in fast epithelialization and wound healing [25], thus, improving pain, through use of less analgesics, low rate of immobilization, and resulted in the earlier discharge of patients. Amniotic membrane is rich in collagen and several growth factors that support the healing process to both advance wound closure and diminish scar formation. Its distinctive properties include the lack of immunologic markers, antibacterial properties, and the ability to reduce pain on application. Recently, techniques have been developed to dehydrate the amnion while preserving many of these wound-healing attributes, to produce a temperature-stable allograft [26].

## 6. Newer Approaches for Tissue Engineering

Different strategies, such as injecting growth factors and extracellular matrix, are being adopted towards tissue re-growth and wound healing. Some of the recent strategies are listed below.

### 6.1. Cell Cocultures

Cell-based approach to develop skin substitutes usually involves differentiated, embryonic, or induced pluripotent stem cells. Cells such as human dermal fibroblasts [27], foreskin derived keratinocytes [28], keratinocyte stem cells [29], hair follicle stem cells [30], angiogenic endothelial progenitor cells [31], bone marrow-derived mesenchymal stem cells [32], and adipose tissue derived mesenchymal stem cells [33] are mainly used for wound healing and tissue regeneration. Work is progressing toward co-culturing cells for tissue generation that involves keratinocytes and fibroblasts [34,35]. Keratinocyte sheets of stratified epithelia can be reconstructed from human epithelial cells [36,37]. Moreover, cultured dermal substitutes containing fibroblasts can be added to keratinocytes sheets. Fibroblast cells can stimulate keratinocyte growth and differentiation by either secreting soluble growth factors or via cell–cell contacts and cell matricial element contacts. In turn, keratinocytes can positively affect fibroblast proliferation [38]. Dermal fibroblasts are also important in skin remodeling and acute wound contraction [39]. Melanocytes and Langerhans’ cells can also be cultured along with keratinocytes and fibroblasts. Melanocytes can recreate the natural pigmentation process whereas Langerhans’ cells monitor skin immunological reactions. Recently, complex three-dimensional (3D) models are being developed using these co-cultured cells with the goal of making engineered tissues similar to their natural counterparts. An exclusive full-thickness 3D skin equivalent was developed to model early melanoma invasion by incorporating an inert scaffold with suitable pore sizes to support the 3D development and cell–cell interaction of primary human dermal fibroblasts [40].

### 6.2. Cultured Epithelial Autografts

Epithelial autografts are used in burn care [41,42] and were first reported in clinical use in 1981. These autografts can be constructed using keratinocytes. Keratinocytes were first cultured successfully in the laboratory about 30 years ago [43,44]. Keratinocyte cells can be generated from a small biopsy of healthy skin, expanded into sheets for few weeks, which is then co-cultured on mouse fibroblasts [45,46]. Epithelial autografts are, however, limited due to difficulty of handling, unpredictable uptake, and high cost, and thus need a delivery system or a support dressing.

### 6.3. Tissue Engineered Skin Substitutes

Due to deficiency of donor skin graft supplies, tissue-engineered skin substitutes present an efficient alternative in substituting donor skin grafts. Skin substitutes are emerging therapeutic tools with a wide range of applications. Skin substitutes act as a temporary protective cover of the wound bed, thus protecting damaged regions from fluid loss and contamination along with accelerating the wound-healing processes by promoting release of cytokines and growth factors at the wound site [47].

An absorbent dressing made of cotton wool sandwiched between layers of gauze was described by Joseph Gamgee in 1880 and used as “skin substitutes” for treating wounds [48]. Likewise, in 1895, Mangoldt described a technique of “epithelial cell seeding” for treating chronic wounds [45]. His technique involved harvesting epithelial cells from superficial epithelium of skin and seeding them onto the wounds [49]. Similarly, in 1897, Lunggren kept skin fragments alive by inoculating them in ascitic fluid at room temperature [45,50]. Likewise, keratinocytes were grown successfully on lethally irradiated murine fibroblasts [43,45]. Cultured autologous epithelium was used to cover burn defects for the first time by O’Conner [42,48]. Likewise, a dermal substitute was developed based on collagen gel with fibroblasts to which an epidermal layer was added, thus making a “skin equivalent”, “composite culture”, or “organotypical culture” [45,50].

**Figure 2:**
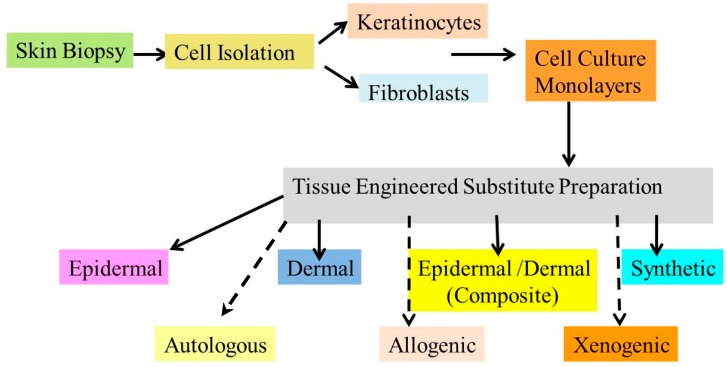
Tissue engineered skin substitute preparation. Bold lines indicate cell type for tissue engineered substitute and dotted lines indicate cell source.

Tissue engineered skin substitute preparation involves cells and/or extracellular matrix (ECM) [18] (Figure 2). An ideal synthesized skin substitute should be sterile, act like a barrier, have low inflammatory response, provide no local or systemic toxicity, and allow water vapor transmission similar to normal skin. These skin substitutes should also adhere to wound surface rapidly, have required physical and mechanical properties, and go through controlled degradation [51]. They should also be easy to handle, agile to conform to irregular wound surfaces, relatively inexpensive, and facilitate angiogenesis. They should also have a long shelf-life with low storage requirements and stress resistance in engineered skin substitutes [52]. When biomaterials are used for substitutes, they should be biodegradable, repairable, non-toxic, non-immunogenic, and non-inflammatory with low risk of disease transmission. Easy availability, long-shelf life, and user-friendliness makes an ideal engineered substitute. A suitable biomaterial is crucial in the development of functional engineered tissues. Different approaches have been adopted to develop engineered tissues, such as synthetic membranes for mono- or multi-layered cultures and 3D matrices for full-thickness models. Properties of the “ideal” skin substitute for in vivo use have recently been reviewed by MacNeil [51].

Tissue engineered skin provides both epidermal and dermal components required to achieve functional wound closure and have therefore been used to effectively close full-thickness burn wounds and treating burns that are greater than 50% of the total burn surface area (TBSA) [7,46,53,54]. The presence of a large number of cells, especially stem cells, in tissue engineered skin enables regeneration of native-like skin in burn patients.

Tissue engineered skin is mainly of three types: (a) cultured epidermal cells with no dermal components; (b) with only dermal components; or (c) a bilayer containing both dermal and epidermal components [55]. Although, each of these has their own advantages and applications, none of them can fully simulate native skin.

## 7. Types of Skin Substitutes

Currently, available skin-substitutes can be classified in different ways [4,49,51,55,56,57]. Based on duration of cover, they are classified as permanent, semi-permanent or temporary. Based on anatomical structure, they are classified as epidermal, dermal, or dermo-epidermal (composite). They can also be classified based on skin substitute composition as cellular or acellular. Similarly, based on the type of the biomaterial used, they can be biological (autologous, allogeneic, xenogeneic) or synthetic (biodegradable, non-biodegradable).

Synthetic skin substitutes are made up of acellular materials. They are designed primarily to function as barriers to fluid loss and microbial contamination. Commonly used synthetic acellular skin substitutes are Biobrane^®^, Integra^®^, Alloderm™, and TransCyte™. The natural skin substitutes, commonly called tissue engineered skin, are mainly cultured allogenic or autologous cell suspensions or sheets, which are used on their own or along with a dermal matrix. Examples of frequently used natural skin substitutes with allogeneic cells include Dermagraft™, Apligraf™, and OrCel™, while those with autologous cells include Epicel™ (Table 1). These substitutes are further described below.

### 7.1. Acellular Skin Substitutes

**Figure 3:**
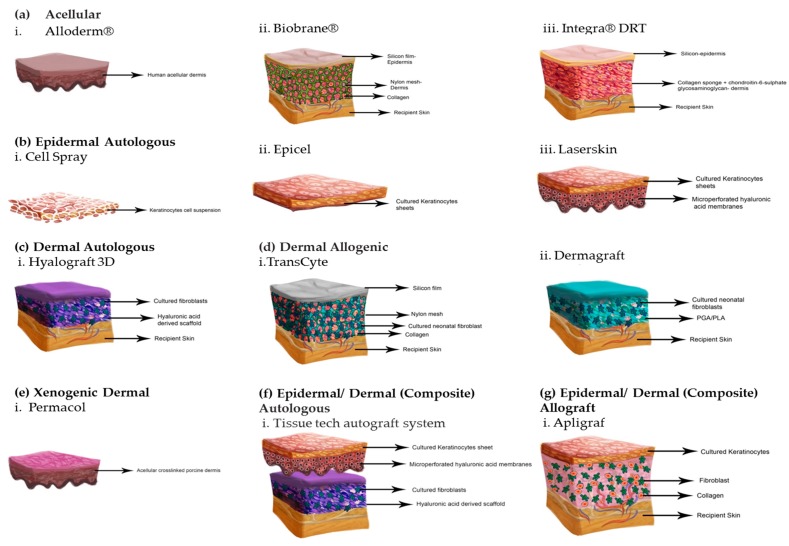
Tissue engineered skin substitutes. (**a**) Acellular: i. Karoderm ii. Biobrane iii. Integra (**b**) Epidermal Autologous: i. Cell Spray ii. Epicel iii. Laserskin (**c**) Dermal Autologous: i. Hyalograft 3D (**d**) Dermal Allogenic: i. TransCyte ii. Dermagraft (**e**) Dermal Xenogenic: i. Permacol (**f**) Epidermal/Dermal (Composite) Autologous i. Tissue tech Autograft system (**g**) Epidermal/Dermal (Composite) Allograft i. Apligraf.

The use of acellular skin substitute started in late 1970s and is used mainly as a temporary skin substitute for superficial or mid-dermal partial thickness wounds and burns. They can also be used for donor sites and congenital diseases, such as epidermolysis bullosa [58,59] and in hydradenitis suppurativa [60]. They usually consist of a nylon mesh or collagen, acting as a “dermis” and a silicon membrane as an “epidermis”. There are three kinds of commercial available acellular skin substitutes: Biobrane^®^, Integra^®^, and Alloderm (Figure 3a). Details about their composition are in Table 1.

### 7.2. Cellular Allogenic Skin Substitutes

Cellular allogenic skin substitutes are mainly produced using living neonatal foreskin fibroblasts with a mesh or a matrix. They have been used successfully in vestibuloplasty, after mucogingival junction and supra-periosteal dissection [46,49]. They have also been used for treatment of venous and diabetic ulcers along with wound management in epidermolysis bullosa, skin cancer, and in burns [46,49]. Examples include Transcyte^®^, Dermagraft^®^, Apligraf^®^, and Graftskin^®^ (Figure 3d,f,g).

### 7.3. Cellular Autologous Skin Substitutes

Acellular or cellular allogenic skin substitutes only provide temporary coverage to raw skin surfaces. These have to be either replaced by a split skin graft or re-grafted in the case of large wounds. In the case of smaller wounds, gradual epithelialization from the wound itself is required.

Cultured autologous keratinocytes provide more permanent skin coverage for various types of wounds. These cells are cultured based on the Rheinwald and Green [43,44] technique. There are two types of cellular autologous skin substitutes available: Cultured Epidermal Autograft (CEA) and Cultured Skin Substitutes (CSS).

CEA involves the culture of autologous keratinocytes, derived from skin biopsy of the patient [45,46]. However, after few weeks, it is challenging to handle these keratinocytes, thus requiring a delivery system or a supporting dressing [48,49]. Commercially available CEAs have different delivery or carrier systems. Keratinocytes alone may not heal full thickness wounds or burns. Another concern with CEA is the growth of blisters with friction, since their dermal epidermal junction is not completely developed. It can also lead to increased scarring, contracture and hyperkeratosis [46]. Furthermore, due to the digestive properties of collagenase enzymes within the wound bed, the uptake of CEA is reduced to about 30%–80%. To overcome the issue of CEA uptake, an alternate method was developed [18,48], which included acclimatization of the wound bed with cadaveric allogenic skin for four days before grafting followed by placing autologous cells after stripping the allo-epidermis [18,48].

CSS is an autologous graft with both epidermal and dermal components. It acts as a permanent coverage with well-formed dermal-epidermal junction and is easy to handle. CSS is expensive and has a longer preparation time. [18,46,49]. Different dermal biosynthetic scaffolds are being used to develop several types of CSS. Hyaluronic acid derived substitute is often used to prepare CSS. These types of CSS can stimulate fibroblasts growth and movement, controls osmoregulation and matrix hydration, scavenge free radicals and regulate inflammation [61,62].

### 7.4. Commercially Available Skin Substitutes

There are many commercial skin substitutes, permanent or temporary, that are available in the market designed for use with specific clinical issues [19,22,51,55,63,64]. Commercially available engineered skin substitutes’ models vary in techniques and cell sources (Figure 3). Table 1 lists some of the commercially available skin substitutes.

Skin substitutes like Biobrane^®^, Integra^®^, and Alloderm are made of acellular material, such as a nylon mesh or collagen (Figure 3a). Biobrane^®^ and Integra^®^ are synthetic bilayer skin substitutes. Biobrane^®^ is made of a nylon mesh mimicking as a “dermis” and a silicone membrane as an “epidermis” implanted in porcine collagen [46,49,55]. Integra^®^ consists of a silicone membrane as an epidermal layer and dermal layer made of bovine collagen and shark chondroitin-6-sulphate glycosaminoglycan [18,46,49]. Although, Biobrane is used for covering partial thickness burns in a single stage procedure, it has the drawback of being intolerant to contaminated wound beds. Integra, on the other hand, has good long term aesthetic and functional outcome, but is a two stage process with high cost and poor adhesion [97,98,99,100]. Integra may also include a high vulnerability to infection, graft loss and fluid entrapment, requiring fenestration of the tissue. Another substitute that falls in this category is Advanced Wound Bioengineered Alternative Tissue (AWBAT) [22,101], which is made of porous silicone membrane bonded with a continuous 3D nylon structure that contains non cross–linked porcine type 1 collagen peptides.

Alloderm, Graftjacket, and GammaGraft are allografts derived from an acellular matrix obtained from a cadaveric dermis [22]. As a decellularized tissue-engineered skin substitute, alloderm consist of a cell-free matrix incorporated into the wound bed [65]. It has a basement membrane but lacks an epidermal layer. The acellular matrix provides a good natural medium for fibroblast and endothelial cells to regenerate from the neoderm [18,46,49]. It is immunologically inert and offers natural dermal porosities for renewal and vascularization on the wound bed, but has the drawback of being a two stage very expensive technique with a risk of transmitting disease [51,91,102,103].

Other commercial skin substitutes such as Apligraf^TM^, Celaderm^TM^, Dermagraft^TM^, Trancyte^TM^, and OrCel^TM^ (Figure 3), are constructed from sheets of cells derived from neonatal (allogenic) foreskin [19,63,92,93]. TransCyte™ has similar configuration to Biobrane^®^ and consists of a nylon mesh seeded with fibroblasts cultured from neonatal human foreskin. The foreskin secretes ECM components and growth factors for efficient tissue regeneration. It is often used as a temporary cover for excised burns and considered more beneficial for wound healing compared to strictly synthetic skin substitutes [81,104]. Transcyte has the advantage of immediate availability and easy storage but is only a temporary solution.

Dermagraft, on the other hand, is an allogeneic dermal substitute created by the combination of living neonatal foreskin fibroblasts cells and biodegradable mesh from polyglycolic acid biomaterials [105]. The fibroblasts are cryopreserved at −80 °C and when implanted to the wound, they regain their viability and proliferate and produce growth factors and ECM components [106]. The polyglycolic acid mesh is absorbed within 3–4 weeks [46,49]. Dermagraft is easy to handle with coverage to chronic wounds and diabetic ulcers with no rejection. However, it lacks strong ECM structure leading to infections and cellulitis [51,98,103].

Skin substitutes of composite allograft category include Apligraf^TM^ and Orcel^TM^. Apligraf^TM^ contain both living dermis and epidermis and is formulated by mixing living fibroblasts from neonatal foreskin with bovine collagen. This mixture is further exposed to heat to produce a loose matrix, which develops a dermal fibrous network. Cells are proliferated on the dense fibrous matrix [61]. In the treatment of venous leg ulcers and diabetic foot ulcers, Apligraf™ is often used [61]. Treating wounds with Apligraf for more than four weeks results in enhanced healing when compared with any other skin substitute currently available in the market. The downside of Apligraf is that it is rather expensive, with a short shelf-life (range: 5–10 days), and although the risk is significantly low, there is a likelihood of disease transmission owing to Apligraf being allogenic in nature [107,108]. Similar to Apligraf^TM^, OrCel^TM^ is another skin substitute that is made up of fibroblasts seeded into a bovine collagen type I matrix (dermal side) and keratinocytes cultured at the air–liquid interface (epidermal side) [86,87,88]. It provides a favorable matrix for host cell migration and is used for grafting onto partial-thickness wounds; nonetheless, there is an amplified risk of rejection and disease due to the presence of the bovine collagen [52,107].

Other skin substitutes like Permacol, Matriderm, and Oasis belong to the xenograft category [79,85]. Permacol is made of porcine dermis, whereas Matriderm^®^ is made of a matrix of bovine type I collagen with elastin (Figure 3e). It is utilized for dermal regeneration. With the progression of healing process, fibroblasts lay down the ECM and the Matriderm^®^ resorbs [109]. Permacol has a good aesthetic and functional outcome but is prone to infection, hematomas, and seromas [73,74], whereas, Matriderm involves a single stage procedure with increased vascularization and elasticity of regenerating tissue but lacks enough scientific evidence to verify its efficacy of a one-step procedure [103,110,111]. Another substitute is Oasis, which is derived from porcine intestinal submucosa [79,85].

The commercially available CAE substitutes, such as Epicel™, are made of autologous keratinocytes sheets attached to a petrolatum gauze support, which is detached approximately one week after grafting [73]. Epicel™ is used on patients with full-thickness burns covering more than 30% total body surface area (TBSA). It is also used on patients with giant congenital nevus [73]. Epicel, however, has a longer generation time. It takes almost three weeks to culture the tissue for use. Since the tissue is an autograft, there is a very low risk of rejection [107,112].

Another variation of CAE substitutes is a suspension, such as Cell Spray (Figure 3b). Keratinocytes harvested in a suspension from a split-thickness donor biopsy is placed in the wound to create an epidermal cover. As the application is aerosolized, it allows complete coverage of contoured wounds [70,71,72,79]. The downside of this application is that there is a risk of infection or donor rejection [77,78,79].

Epidex is an additional CAE substitute, having small keratinocyte sheets cultured from patient’s follicles [67]. It can be expanded in vitro for large burn areas. Though it is a permanent substitute, it is also fragile and expensive [41,42]. Like Cell Spray CAE, subconfluent cells on a carrier make epithelial substitutes, such as Myskin (CellTran) [68].

Hyalograft 3D is another autologous dermal substitute. In this product, autologous cultured fibroblasts are seeded onto a 3D hyaluronic acid derived scaffold [79]. Hyalomatrix^®^ is a bilayer hyaluronan base scaffold with autologous fibroblasts and an outer silicone membrane (Figure 3c). The silicone membrane acts as a temporary epidermal barrier and hyaluronan is delivered to the wound bed [84]. Laserskin is another autologous epidermal substitute [74]. Another similar substitute is TissueTech autograft system, which has been used in successful treatment of diabetic foot ulcers (Figure 3f) [80]. It combines two tissue-engineered biomaterials, a dermal replacement construct Hyalograft 3D and an epidermal substitute Laser skin [80]. In this system, autologous keratinocytes and fibroblasts are grown on micro perforated hyaluronic acid membranes. Up to 70.3% of wound closure with an area greater than 5 cm^2^ in 85% of cases was accomplished in many full-thickness ulcers using TissueTech autograft system [80]. The rate of recurrence was also low with this TissueTech Autograft System. Although this system may allow for absolute wound closure, it is not a “rue” bilayered skin substitute where both dermal and epidermal layers are present, as it entails grafting of two products and may be difficult to use in a clinical setting [80].

Other skin substitutes are based on fibroblasts seeded onto a synthetic polymer membrane [89], such as polylactic-glycolic acid (PLGA), polycaprolactone (PCL), a combination of PLGA/PCL, and mixtures made of hyaluronic acid (HA). Polypyrrole (PPy) can be used as a cultured dermal substitute [83]. PPy and HA, in particular, are reported to promote skin regeneration and cell growth. Polypyrrole (PPy) has good in vitro and in vivo biocompatibility and can be fabricated with a large surface area, with different porosities. However, PPy is very difficult to further process once synthesized, as its molecular structure makes it non-thermoplastic, mechanically rigid, brittle and insoluble after synthesis [113,114,115].

An allogenic living epidermal substitute such as Stratagraft is a full thickness skin substitute consisting of human dermal fibroblasts as a dermal component and neonatal keratinocytes as a fully-stratified epidermis [79,94,95,96]. It serves as a bridge before autografting in burn patients and other severe skin wounds. Stratagraft is well-tolerated and is not acutely immunogenic in patients with traumatic wounds [87].

Alternative skin substitute Permaderm™ contains both epidermal and dermal components composed of autologous fibroblasts and keratinocytes cultured on a collagen substrate. It has the advantage of being a one-step procedure with permanent replacement of dermal and epidermal layers. However, no clinical trials have been reported yet [46,116,117,118,119,120]. Another full thickness skin substitute that is used in chronic therapy-resistant leg/foot ulcers is Tiscover™. Similarly, DenovoDerm™ and DenovoSkin™ are also full-thickness dermal skin substitutes that are currently undergoing trials. Denovoskin has a near normal skin architecture but has long culture times with no clinical series yet reported [7,51]. However, each of these products has its own limitations, and there is no perfect or ideal skin substitute yet [78].

## 8. Limitations of Commercially Available Skin Substitutes

There are several limitations to the commercially available skin substitutes, like reduced vascularization, poor mechanical integrity, failure to integrate, scarring, and immune rejection [51]. Due to the inability to revascularize, cells in the substitute die and slough away from the host tissue. Few commercially available skin substitutes allow limited vascularization. Moreover, the development of engineered skin is a time consuming process involving 2–3 weeks of cell culture before it is ready for grafting. Technical advances in cell and tissue culture protocols are required to overcome cell growth issues of skin substitutes. Currently, available skin substitutes mainly consist of fibroblasts and keratinocytes and therefore lack the ability to make differentiated structures, like hair and sweat glands. Therefore, there is a need to include additional cells types, such as endothelial cells in engineered skin. There is also a need to reduce the cost of current skin substitutes. The cost to cover 1% body surface area with Epicel™ is more than $13,000 [78,121]. In order to meet the massive demand for skin substitutes from hospitals for clinical applications, it is necessary to improve large-scale production.

## 9. Future Perspectives

Tissue engineered skin substitutes hold a promise for future tissue regeneration and wound healing therapeutics. There is still a need for improvement in vascularization of these substitutes to increase their life span and provide better integration with host tissues. One way of increasing vascularization is by using bioreactors to provide mechanical stimulation necessary to develop mature blood vessels [122]. An important step in developing reliable substitutes will be to standardize the production process and reduce manufacturing costs. Moreover, the standardization of storage and preservation is also important to extend their life spans. Further investigation is also required to assess the possibility of increased risk of future malignancies in such cells. One way to eradicate these issues will be to mimic more properties of in vivo skin. Although there is an urgent need to improve tissue engineered skin substitutes due to practical and therapeutic limitations, the field has come a long way in patient healing and does hold promise in the near future for skin and wound healing. Better and efficient products can be developed by a detailed understanding of the mechanism of the therapeutic action of bioengineered skin. There are several skin substitutes currently available, but skin substitutes constructed from a combination of stem cells and biomaterials remain a promising solution for the future.

## Figures and Tables

**Table 1:** Commercially available skin substitutes.

Substitute Type	Product	Company	Components	References
Acellular	Alloderm^®^	LifeCell Inc., Branchburg, NJ, USA	Human acellular lyophilized dermis	[18,22,46,49,65]
	SureDerm	HANS BIOMED Corporation, Daejeon, Korea	Human acellular lyophilized dermis	-
	OASIS Wound Matrix	Cook Biotech Inc., West Lafayette, IN, USA	Porcine acellular lyophilized small intestine submucosa	[66]
	Biobrane^®^	Mylan Bertek Pharmaceuticals, USA	Ultrathin silicone as epidermal analog film and 3D nylon filament as dermal analog with type I collagen peptides	[46,49,55]
	Integra^®^ DRT (dermal regeneration template)	Integra^®^ LifeSciences Corp., USA	Dermal analog—bovine collagen and chondroitin-6-sulfate GAG; epidermal analog—silicone polymer polysiloxane	[18,46,49,55]
**Epidermal**				
Autologous	EpiDex	Modex Therapeutiques, Lausanne, Switzerland	Cultured keratinocytes from outer root sheath of scalp hair follicles (confluent cell sheet)	[67]
	EPIBASE	Laboratoires Genevrier, Antibes, France	Cultured keratinocytes (confluent cell sheet)	-
	MySkin	CellTran Ltd., UK	Cultured keratinocytes (subconfluent cell sheet) silicone support layer with a specially formulated surface coating	[68]
	Bioseed-S	BioTissue Technologies GmbH, Germany	Cultured keratinocytes (subconfluent cell suspension) fibrin sealant	[69]
	CellSpray	Clinical Cell Culture (C3), Australia	Non-/cultured keratinocytes (subconfluent cell suspension)	[70,71,72]
	Epicel^®^	Genzyme Biosurgery, USA	Sheets of autologous keratinocytes attached to petrolatum gauze support	[73]
	Laserskin^®^ or Vivoderm	Fidia Advanced Biopolymers Srl, Italy	Autologous keratinocytes and fibroblasts, grown on microperforated hyaluronic acid membranes	[74]
	Autoderm (Autologous Inferior Dermal Sling)	XCELLentis NV, Belgium	Cultured keratinocytes	[75]
	TransDerm	XCELLentis NV, Belgium	Cultured keratinocytes	[75]
	Lyphoderm	XCELLentis NV, Belgium	Lyophilized neonatal keratinocytes	[76]
	Cryoceal	XCELLentis NV, Belgium	Cryopreserved keratinocytes	[77]
**Dermal**				
Autologous	denovoDerm™	EUROSKINGRAFT, Switzerland	Autologous dermal substitute	[78]
	Pelnac Standard/Fortified	Gunze Ltd., Japan	Porcine tendon derived atelocollagen type I, sponge layer with silicone film	-
	Hyalomatrix PA	Fidia Advanced Biopolymers, Italy	HYAFF (an ester of hyaluronic acid) layered on silicone membrane	-
	Hyalograft 3D	Fidia Advanced Biopolymers, Italy	Cultured fibroblasts hyaluronic acid membrane (HAM)	[79,80]
Allogenic	Dermagraft^®^	Advanced BioHealing, Inc., USA	Bioabsorbable polygalactin mesh matrix seeded with human neonatal fibroblasts and cryopreserved	[78]
	TransCyte^®^	Advanced BioHealing, Inc., USA	Collagen-coated nylon mesh seeded with allogenic neonatal human foreskin fibroblasts	[81]
	Terudermis	Olympus Terumo Biomaterial Corp., Japan	Silicone, bovine lyophilized crosslinked collagen sponge made of heat-denatured collagen	[82]
	Cyzact (ICX-PRO)	Intercytex, St John’s Innovation Center, UK	Cultured allogeneic human dermal fibroblasts embedded in a human fibrin gel matrix	-
	ICX-SKN skin graft replacement	Intercytex, St John’s Innovation Center, UK	Cultured dermal fibroblasts natural human collagen matrix	-
	Polycaprolactone collagen nanofibrous membrane	National University of Singapore, Singapore	Cultured dermal fibroblasts polycaprolactone-blended collagen electrospun nanofibrous membrane	[83]
	Tegaderm-nanofibre construct	National University of Singapore, Singapore	Cultured dermal fibroblasts poly(e -caprolactone)/gelatin nanofibrous scaffold electrospun on polyurethane dressing	-
	Collagen–glycosaminoglycan–chitosan dermal matrix seeded with fibroblasts	INSERM, France	Cultured dermal fibroblasts bovine collagen I/chondroitin-4/6-sulfate/chitosan lyophilized dermal matrix	[46,49,55]
	Human hair keratincollagen sponge	Southern Medical University, China	Cryomilled porcine acellular diisocyanite cross-linked dermis	-
	Hyaluronan-FNfds hydrogel matrix	SUNY at Stony Brook, USA	Hyaluronan coupled with fibronectin functional domains	[84]
	Composite nano-titanium oxide–chitosan artificial skin (NTCAS)	Cardinal Tien College of Healthcare and Management, Taiwan	Composite nano-titanium oxide–chitosan with gelatin and hyaluronic acid	-
Xenogeneic	Permacol Surgical Implant	Tissue Science Laboratories plc, UK	Porcine acellular diisocyanite crosslinked dermis	[79,85]
	Matriderm	Dr Suwelack Skin and HealthCare AG, Germany	Bovine non-cross-linked lyophilized dermis, coated with a-elastin hydrolysate	[79,85]
	EZ DermTM	Brennen Medical Inc., USA	Porcine aldehyde cross-linked reconstituted dermal collagen	[18]
	Bovine collagen cross-linked with microbial transglutaminase	National University of Ireland, Ireland	Freeze-dried bovine collagen scaffold cross-linked with microbial transglutaminase	[86,87,88]
	Collatamp	SYNTACOLL AG, Switzerland	Multilayer bovine collagen matrix	-
Synthetic	Hybrid nanofibrous PLGA/chitosan membrane	Tianjin University, China	PLGA/chitosan hybrid electrospun nanofibrous membrane	[83,89]
	Biodegradable polyurethane microfibers	University of Delaware, USA	Biodegradable polyurethane microfibres	[90]
**Epidermal/Dermal (Composite)**				
Autologous	Permaderm™ (Cincinnati Shriners Skin Substitute)	Regeninic Inc.USA	Autologous fibroblasts and keratinocytes in culture with bovine collagen and GAG substrates	[78]
	Tiscover™ (A-Skin)	Advanced Tissue Medicinal Product, Netherlands	Autologous full thickness cultured skin	[78]
	denovoSkin™	EUROSKINGRAFT, Univ. of Zurich, Switzerland	Autologous full thickness substitute consisting of dermal and epidermal layers	[78]
	PolyActive	HC Implants BV, Netherlands	Cultured keratinocytes and fibroblasts polyethylene oxide terephthalate (PEO)/polybutylene terephthalate (PBT)	[91]
	TissueTech Autograft System (Laserskin and Hyalograft 3D)	Fidia Advanced Biopolymers, Italy	Cultured keratinocytes and fibroblasts microperforated hyaluronic acid membrane (HAM)	[74,79]
Allogenic	Apligraf^®^	Organogenesis Inc., USA	Bovine collagen matrix seeded with neonatal foreskin fibroblasts and keratinocytes	[19,63,92,93]
	OrCel^®^	Ortec International Inc., USA	Type I collagen matrix seeded with neonatal foreskin fibroblasts and keratinocytes	[86,87,88]
	Karoskin (Karocells)	Karocell Tissue Engineering AB, Sweden	Native human cadaver skin with dermal and epidermal cells	[55]
	CeladermTM	Celadon Science LLC, USA	Sheets of cells derived from neonatal (allogenic) foreskin	[19,63,92,93]
	StrataGraft™	Stratatech *Corporation*, USA	Full thickness skin substitute with dermal and fully differentiated epidermal layers	[79,94,95,96]
	AcuDress	DFB Pharmaceuticals, Inc., USA	Cultured keratinocytes fibrin substrate	-
	Allox	DFB Pharmaceuticals, Inc., USA	Sprayed suspension of allogeneic keratinocytes and fibroblasts in fibrin substrate	-
Xenogeneic	Oasis^®^	Healthpoint Biotherapeutics, USA	Intact matrix from porcine small-intestine submucosa and intended for wound closure stimulation in acute, chronic and burns wounds	[79,85]
